# Desigualdades en el control odontológico prenatal en Colombia, un análisis a partir del IV Estudio Nacional de Salud Bucal, 2013-2014

**DOI:** 10.7705/biomedica.5705

**Published:** 2021-06-15

**Authors:** Lorena Alexandra Maldonado-Maldonado, Sandra Patricia Misnaza-Castrillón, Carlos Andrés Castañeda-Orjuela

**Affiliations:** 1 Observatorio Nacional de Salud, Instituto Nacional de Salud, Bogotá, D.C., Colombia Instituto Nacional de Salud Bogotá, D.C. Colombia

**Keywords:** salud bucal, atención prenatal, servicios de salud, disparidades en atención de salud, prevención primaria, derecho a la salud, Oral health, prenatal care, health services, healthcare disparities, primary prevention, right to health

## Abstract

**Introducción.:**

La atención odontológica es esencial para garantizar el derecho a una maternidad saludable. En Colombia, las políticas de atención prenatal incorporan la promoción, prevención y atención en salud bucal como parte de la atención integral que deben recibir las mujeres gestantes en el sistema de salud, sin embargo, no se hace un seguimiento sistemático del cumplimiento de estas directrices.

**Objetivo.:**

Explorar la atención efectiva y las desigualdades sociales en la prestación y el uso del control odontológico prenatal en Colombia.

**Materiales y métodos.:**

Estudio descriptivo con datos de las mujeres gestantes reportadas en el Cuarto Estudio Nacional de Salud Bucal, 2013-2014. Se estimaron las desigualdades sociales absolutas y relativas, según zona de residencia, pertenencia étnica, nivel educativo, régimen de afiliación a los servicios de salud y estrato socioeconómico.

**Resultados.:**

Se analizaron los datos de 1.050 mujeres gestantes. El 88,37 % recibió control prenatal y, el 57,19 %, control odontológico. Se observó un patrón general de brechas sociales en el uso efectivo de este último servicio, principalmente en función del aseguramiento. Las mujeres gestantes con mayor posibilidad de recibir atención odontológica prenatal fueron aquellas con algún aseguramiento en salud (razón de prevalencias, RP=2,62; IC_95_% 2,12-3,12), residentes en zonas urbanas (RP=1,37; IC_95_% 1,18-1,56), con nivel educativo técnico o superior (RP=1,20; IC_95_% 1,02-1,38) o de estratos sociales medios o altos (RP=1,15; IC_95_% 1,01-1,29).

**Conclusiones.:**

En Colombia, la prestación efectiva del control odontológico a mujeres gestantes como parte de la atención prenatal integral, sigue siendo un reto. Se requieren importantes esfuerzos para cumplir las normas y reducir las desigualdades sociales en el acceso a este servicio.

## Abstract (en)

**Introduction:**

Dental care is essential in guaranteeing the right to healthy motherhood. In Colombia, prenatal care policies incorporate the promotion, prevention, and care in oral health as part of the comprehensive care that pregnant women must receive within the health system. However, there is no systematic monitoring of compliance with these guidelines.

**Objective:**

To explore effective care and social inequalities in the provision and use of prenatal dental control in Colombia.

**Materials and methods:**

This was a descriptive study with data on pregnant women reported in the Fourth National Oral Health Study, 2013-2014. Absolute and relative social inequalities were estimated according to the area of residence, ethnicity, education level, health affiliation regime, and socioeconomic stratum.

**Results:**

We analyzed the data from 1,050 pregnant women. In total, 88.37% received prenatal control and 57.19%, dental control. We observed a general pattern of social gaps in the effective use of the latter, mainly due to the health insurance regime. The pregnant women with the greatest possibility of having some prenatal dental control, were those with some health insurance (prevalence ratio [RP]=2.62; CI 95%: 2.12-3.12), residents in urban areas (RP= 1,37; CI95%: 1.18-1.56), with higher and/or technical education level (RP=1.20; CI95%: 1.02-1.38) or from mid-high social strata (RP=1.15; CI95%: 1.01-1.29).

**Conclusions:**

In Colombia, the effective provision of dental control to pregnant women as part of comprehensive prenatal care continues to be a challenge. Significant efforts are required to comply with regulations and reduce social inequalities in access to this service.

La maternidad saludable ha sido reconocida a nivel mundial como parte esencial del derecho fundamental a la salud, y como un asunto clave para el desarrollo humano y la superación de inequidades sanitarias (1). En este sentido, los estados deben garantizar una serie de cuidados orientados a proteger la integridad y el bienestar del binomio madre e hijo, y brindar apoyo y seguridad a la mujer en su proceso de gestación.

Entre los cuidados que requieren las mujeres gestantes, debe contemplarse el cuidado de la boca, ya que los cambios fisiológicos, hormonales y nutricionales experimentados durante el embarazo contribuyen a la aparición de afecciones bucales que pueden tener serias implicaciones para la madre y su hijo en gestación. En los estudios se ha evidenciado que las afecciones periodontales durante el periodo gestacional se correlacionan con la presencia de preeclampsia, diabetes mellitus gestacional, parto prematuro, bajo peso al nacer y aumento en la hospitalización de los neonatos en unidades de cuidados intensivos (2,3). Además, han coincidido en que las afecciones bucales maternas aumentan el riesgo de caries en la primera infancia y la adolescencia (4,5), lo que se considera de importancia en salud pública debido al impacto negativo en el sano crecimiento y desarrollo de los niños, y a los costos que genera tanto para las familias como para los sistemas de salud (5).

Actualmente, la persistencia de desigualdades en la salud bucal de las mujeres gestantes debidas a su nivel socioeconómico, su pertenencia étnica, su zona de residencia (urbana o rural) y su grupo etario, han llevado a un consenso a nivel mundial sobre la necesidad de implementar acciones para reducir las brechas en el estado de la salud bucal de los diferentes grupos sociales dentro y entre países. Las acciones de promoción de salud y prevención de las enfermedades bucales antes y durante la gestación se plantean como una de las intervenciones más costo-efectivas para preservar la salud y la calidad de vida de la mujer y de su hijo (6), así como para reducir inequidades sociales en lo tocante a la salud bucal de madre e hijo (7,8). Estas intervenciones son responsabilidad de las mujeres, su familia, la comunidad, los profesionales de la salud, los medios de comunicación y el Estado (5,9).

En Colombia, la situación de la salud bucal de las gestantes es un asunto que requiere más visibilidad y atención. Según el IV Estudio Nacional de Salud Bucal - ENSAB IV, la prevalencia de caries cavitada en mujeres gestantes es del 57,5 %, siendo las menores de 18 años y aquellas que no cuentan con aseguramiento las más afectadas, con una prevalencia de 78,2 y 71,28 %, respectivamente. En cuanto a las afecciones de los tejidos de soporte dental, las superficies dentales comprometidas periodontalmente corresponden al 64,4 % de las mujeres gestantes de 35 a 44 años, al 68,4 % de las de 20 a 34 años y al 75,12 % de las de 18 años (10).

Según el Estado colombiano, las mujeres gestantes son un grupo de especial interés en las políticas públicas, por lo que se han desplegado importantes esfuerzos para mejorar la calidad, oportunidad y gestión de los servicios sanitarios para esta población, con especial énfasis en las intervenciones de protección específica y detección temprana (11,12), incluso desde el momento de las consultas previas a la concepción (13). Como parte de este conjunto de intervenciones, las acciones para mantener una óptima salud bucal han sido constantemente actualizadas en las directrices técnicas y operativas de las políticas (12,14).

Entre las intervenciones en salud bucal incluidas en el plan de beneficios o paquete de servicios garantizados por el sistema de salud, está el control odontológico prenatal, un servicio pensado para generar un diálogo entre la madre y el profesional en odontología, con el fin de visibilizar la importancia de la salud bucal en la gestación, aclarar mitos relacionados con el embarazo y la salud bucal, implementar estrategias de promoción y prevención, y detectar oportunamente afecciones bucales en etapas incipientes para adoptar un plan de tratamiento (15,16).

Aunque desde el año 2000, con la adopción de la Resolución 412, el conjunto de intervenciones materno-perinatales, incluida la atención odontológica prenatal, se consideran actividades de demanda inducida de obligatorio cumplimiento que se deben garantizar gratuitamente a todas las mujeres gestantes en el marco del aseguramiento en salud, no son claros los mecanismos de control para el cumplimiento de estas normas, y en diversos estudios se han evidenciado fallas en el acceso, la integridad y la continuidad de la atención de las mujeres gestantes (17,18).

Dada la importancia de la salud bucal y del control odontológico prenatal en la garantía del derecho fundamental a la salud y a una maternidad saludable, en este estudio se planteó como objetivo explorar la prestación efectiva del control odontológico prenatal en mujeres gestantes colombianas y las desigualdades sociales que las afectan, a partir de fuentes de información secundarias oficiales.

## Materiales y métodos

Se hizo un estudio descriptivo de fuente secundaria sobre la prestación efectiva del servicio de atención odontológica prenatal a mujeres gestantes en Colombia y se exploraron posibles desigualdades sociales en su uso a partir de los microdatos del Cuarto Estudio Nacional de Salud Bucal (ENSAB IV), 2013-2014.

El ENSAB IV fue un estudio poblacional con un diseño muestral probabilístico, estratificado, de etapas múltiples y con reposición de elementos que incluyó a las mujeres gestantes. Se estratificó a nivel de subregión considerando el índice de necesidades básicas insatisfechas (NBI) y la proporción según el tipo de aseguramiento en el Sistema General de Seguridad Social, utilizando la técnica de análisis de conglomerados de k medias.

Para el cálculo de los factores de expansión, se construyeron los inversos multiplicativos de las probabilidades de inclusión o selección según la etapa del muestreo, y el producto de esos factores generó el factor básico de expansión. Además, se ajustó por no respuesta o cobertura, y se construyó un factor de ajuste por totales y por estructura de la población (19).

El ENSAB IV alcanzó un nivel de respuesta general del 88,2 %, con representatividad a nivel nacional y regional; asimismo, brindó información clínica y de algunos factores sociales determinantes a nivel poblacional, e incluyó a mujeres gestantes entre los 12 y los 49 años (19).

Para dar cuenta de la prestación efectiva del control odontológico prenatal, se calcularon tres indicadores: proporción de mujeres gestantes remitidas a odontología por sus servicios de salud, proporción de las remitidas a odontología con asignación de la cita, y proporción de quienes recibieron atención odontológica. Asimismo, a partir de la información disponible en el ENSAB IV, se determinaron las principales razones referidas por las mujeres gestantes para no haber asistido al control odontológico.

Las variables socioeconómicas exploradas en el análisis de desigualdades sociales en la prestación efectiva del servicio de atención odontológica prenatal, fueron zona de residencia (rural o urbana), pertenencia étnica, nivel educativo, régimen de afiliación a los servicios de salud y estrato socioeconómico. El régimen de afiliación a los servicios de salud se refiere al nivel de aseguramiento de las personas en el sistema de salud.

En Colombia, existen fundamentalmente tres regímenes: contributivo, para las personas con capacidad de pago que aportan al sistema; subsidiado, para las personas sin capacidad de pago que reciben subsidios del Estado, y el régimen de excepción para grupos especiales que tienen esquemas propios de seguridad social, como las fuerzas militares y de policía, el magisterio y los empleados de las universidades públicas y de la Empresa Colombiana de Petróleos (Ecopetrol) (20).

El estrato socioeconómico corresponde a una clasificación de grupos de viviendas en función de características como el acceso a los servicios públicos, carreteras y medios de transporte y el valor comercial de la tierra. Esta clasificación, que va desde 1 (más bajo) a 6 (más alto), se utiliza principalmente para definir la asignación de subsidios en el pago de los servicios públicos. En el ENSAB IV estaban disponibles cuatro categorías, las cuales se emplearon en este estudio: muy bajo (1), bajo (2), medio (3) y alto (4 al 6).

Las desigualdades en la prestación del servicio de control odontológico prenatal se calcularon mediante la medición de las brechas de desigualdad absolutas y relativas, tomando como variable dependiente la prestación efectiva de alguna atención odontológica prenatal y, como variables independientes, los indicadores socioeconómicos previamente descritos.

El grupo de referencia en cada variable independiente fue aquel en el que la prevalencia de mujeres gestantes que recibieron algún control odontológico fuera menor. Las desigualdades absolutas se calcularon como la diferencia entre la prevalencia de mujeres gestantes que recibieron algún control odontológico prenatal en cada grupo y la prevalencia del grupo de referencia, en tanto que las relativas se calcularon como la razón entre la prevalencia de mujeres gestantes que recibieron algún control odontológico en cada grupo y la prevalencia del grupo de referencia. Se reportan los intervalos de confianza del 95 % de las diferencias relativas para evaluar la significación estadística de la desigualdad.

Los indicadores obtenidos se ajustaron por los factores de expansión correspondientes y se usó la misma población muestral como denominador. La información se sistematizó y se procesó en Excel 2016™ y en el lenguaje de programación R, versión 3.6.3.

Este estudio no implicaba riesgos para los participantes (21), incorporó en su diseño los principios de beneficencia y no maleficencia (22) y, además, sus resultados pueden aportar al fortalecimiento de las políticas públicas de atención materno-perinatal.

## Resultados

Se analizaron los datos de 1.050 mujeres gestantes reportados en el ENSAB IV a nivel nacional. Las características de la población analizada se presentan en el cuadro 1. Del total de mujeres gestantes, el 88,37 % manifestó haber asistido a cita de control prenatal y el 57,19 % a cita de control odontológico, durante el embarazo. De las mujeres gestantes que asistieron a control prenatal, el 72,27 % se remitieron a odontología, y el 64,57 %, se remitieron y se les asignó la cita con el odontólogo. Las mujeres que se encontraban en su primer trimestre de gestación y que asistieron a control prenatal, tuvieron menos remisiones a odontología (60,58 %), citas asignadas (53,69 %) y controles odontológicos efectivos (39,01 %) (cuadro 2). Tanto el primer control prenatal como el primer control odontológico ocurrieron principalmente en el primer trimestre de gestación, con 71,07 y 31,96 %, respectivamente (cuadro 3).

La región con más controles odontológicos prenatales fue la Oriental, con 65,57 %, y la que menos controles reportó fue la región Pacífico (53,93 %). En las grandes ciudades, el mayor reporte se registró en Medellín (72,60 %) y, el menor, en Bogotá D.C. (51,09 %) (cuadro 4).

De las mujeres gestantes que no asistieron a control odontológico, el 40,68 % adujo no haber sentido la necesidad, el 11,24 %, falta de tiempo, el 6,66 %, miedo o pena, y el 6,64 %, no haber tenido a dónde acudir.

**Cuadro 1:** Características de las mujeres gestantes reportadas en el ENSAB IV, 2013-2014

Variables	n	(%)^a^
Edad (años)		
12 a 19	105	11,28
20 a 29	676	63,00
30 a 39	246	23,05
40 a 49	23	2,65
**Región**		
Atlántica	208	21,89
Oriental	153	15,82
Central	148	14,10
Pacífico	177	16,02
Bogotá	186	15,14
Orinoquia - Amazonia	178	16,99
**Zona de residencia**		
Urbana	899	84,42
Rural	151	15,57
**Nivel educativo**		
Universitario	113	8,88
Técnico	228	21,65
Secundaria	570	55,39
Primaria o menos	139	14,06
**Nivel socioeconómico**		
Medio-alto	189	17,21
Bajo	421	38,18
Muy bajo	440	44,60
**Régimen de afiliación**		
Contributivo	372	35,24
Subsidiado	600	58,93
De excepción	27	1,97
No asegurado	51	3,84
**Pertenencia étnica**		
Mestizo o blanco	704	65,93
No definido	143	14,24
Afrocolombiano	125	12,40
Indígena	57	4,67
Otras	21	2,73

a Porcentajes ajustados con factores de expansión final reportados en el ENSAB IV Fuente: cálculos propios a partir de microdatos del ENSAB IV, 2013-2014

**Cuadro 2:** Prevalencia de controles prenatales, odontológicos y remisiones a control odontológico en mujeres gestantes colombianas, ENSAB IV, 2013-2014

Trimestre de gestación	Mujeres gestantes		Con control prenatal^a^		Remitidas a odontología^b^		Remitidas a odontología con asignación de cita^b^		Con control odontológico^a^	
	n	(%)^c^	n	(%)^c^	n	(%)^c^	n	(%)^c^	n	(%)^c^
Primero	293	26,28	204	71,80	125	60,58	110	53,69	111	39,01
Segundo	399	39,02	368	91,71	274	72,43	240	64,76	241	59,43
Tercero	358	34,70	351	97,14	280	78,64	252	70,45	252	68,44
Total	1.050	100,00	923	88,37	679	72,27	602	64,57	604	57,19

a Prevalencia del total de mujeres gestantes b Prevalencia en grupo de mujeres con control prenatal c Porcentajes ajustados con los factores de expansión final reportados en el ENSAB IV Fuente: cálculos propios a partir de microdatos del ENSAB IV, 2013-2014

**Cuadro 3:** Trimestre de primer control prenatal y primer control odontológico en mujeres gestantes colombianas, ENSAB IV, 2013-2014

Trimestre	Primer control prenatal		Primer control odontológico prenatal	
	n	(%)^a^	n	(%)^a^
Primero	758	71,07	351	31,96
Segundo	151	16,02	218	21,70
Tercero	14	1,28	35	3,53
No reporta	127	11,63	446	42,81

a Porcentajes ajustados con factores de expansión final reportados en el ENSAB IV Fuente: cálculos propios a partir de microdatos del ENSAB IV, 2013-2014

**Cuadro 4:** Controles odontológicos prenatales por región y grandes ciudades - Colombia, ENSAB IV, 2013-2014

**Nivel**	Total de mujeres gestantes (n)	Porcentaje de mujeres gestantes remitidas a odontología (%)^a^	Porcentaje de mujeres gestantes que recibieron control odontológico (%)^a^
Región
Oriental	153	68,87	65,57
Central	148	65,85	58,99
Atlántica	208	65,38	58,29
Orinoquia - Amazonia	178	60,84	54,99
Pacífico	177	60,54	53,93
**Grandes ciudades**
Medellín	29	72,60	72,60
Cali	41	71,19	66,67
Barranquilla	37	71,00	59,78
Bogotá	186	63,32	51,09

a Porcentajes ajustados con factores de expansión final reportados en el ENSAB IV Fuente: cálculos propios a partir de microdatos del ENSAB IV, 2013-2014

### Desigualdades en la prestación del servicio de control odontológico prenatal

Se encontró un patrón de gradiente social para todas las variables socioeconómicas, con una mayor prevalencia de mujeres con algún control odontológico prenatal en los grupos considerados menos vulnerables. Las estimaciones mostraron desigualdades absolutas y relativas en la prestación de la atención odontológica prenatal en todos los indicadores socioeconómicos. En las diferencias relativas se evidenció que las mujeres gestantes con aseguramiento en salud tuvieron hasta 2,62 (IC_95_% 2,12-3,12) veces más posibilidad de tener algún control odontológico prenatal que aquellas que no tenían ningún seguro de salud. Asimismo, las mujeres gestantes que residían en zonas urbanas (desigualdad relativa, 1,37; IC_95_% 1,18-1,56), tenían un nivel educativo técnico o superior (1,20; IC_95_% 1,02- 1,38) o pertenecían a estratos sociales medios o altos (1,15; IC_95_% 1,01-1,29), tuvieron más probabilidad de recibir algún control odontológico que aquellas que residían en zonas rurales, tenían un nivel educativo inferior a primaria, o pertenecían a estratos sociales muy bajos (cuadro 5).

**Cuadro 5:** Desigualdades absolutas y relativas en la prestación del control odontológico prenatal - Colombia, ENSAB IV, 2013-2014

Variable	Control odontológico prenatal			Desigualdad absoluta	Desigualdad relativa IC_95%_
	Sí	No	Sí(%)^a^		
Zona de residencia					
Rural	66	85	43,71		
Urbana	538	361	59,84	16,14	1,37 (1,18-1,56)
**Pertenencia étnica**
Otras	9	12	42,86		
Indígena	26	31	45,61	2,76	1,06 (0,49-1,63)
Afrocolombiano	61	64	48,80	5,94	1,14 (0,61-1,66)
Mestizo/blanco	419	285	59,52	16,66	1,39 (0,89-1,89)
No definido	89	54	62,24	19,38	1,45 (0,94-1,96)
**Régimen de afiliación**
No asegurado	12	39	23,53		
Subsidiado	346	254	57,67	34,14	2,45 (1,95-2,95)
Contributivo - de excepción	246	153	61,65	38,12	2,62 (2,12-3,12)
**Nivel educativo**
Primaria o menos	71	68	51,08		
Secundaria	324	246	56,84	5,76	1,11 (0,94-1,29)
Técnico - universitario	209	132	61,30	10,20	1,20 (1,02-1,38)
**Estrato socioeconómico**
Muy bajo	238	202	54,09		
Bajo	248	173	58,91	4,82	1,09 (0,97-1,21)
Medio-alto	118	71	62,43	8,34	1,15 (1,01-1,29)

a Porcentajes ajustados con factores de expansión final reportados en el ENSAB IV Fuente: cálculos propios a partir de microdatos del ENSAB IV, 2013-2014

## Discusión

En Colombia, no todas las mujeres gestantes que asisten a controles prenatales reciben controles odontológicos, especialmente en el primer trimestre de gestación. La prestación efectiva no solo fue menor con respecto al prenatal, también fue desigual según el nivel de afiliación a los servicios de salud, la zona de residencia (rural o urbana), el nivel educativo y el estrato socioeconómico.

Las desigualdades detectadas constituyen un hallazgo sistemático en diversos estudios que abordan los factores sociales determinantes que generan inequidades en el acceso a los servicios de salud bucal en mujeres gestantes (23-26), lo que llama a reflexionar en torno a la vulnerabilidad diferencial y a las garantías reales de acceso a los servicios odontológicos que tiene esta población sujeto de especial protección en las políticas públicas de salud.

A nivel nacional, el aseguramiento ha permitido el avance en la cobertura efectiva de los controles prenatales (27), pero no así la de los controles odontológicos, pese a que hacen parte del conjunto de intervenciones interdisciplinarias integradas en el control prenatal que debe garantizarse gratuitamente a las mujeres aseguradas antes y durante la gestación (12-14).

La existencia de desigualdades en el acceso a la salud bucal según el nivel de afiliación, hace evidente una limitación estructural del sistema de salud colombiano para garantizar a las mujeres gestantes un acceso universal, oportuno e integral a los servicios sanitarios (28,29), particularmente a los servicios de protección específica y detección temprana. Aunque se encontró que el aseguramiento en cualquier régimen de salud aumentaba la probabilidad de acceder a algún control odontológico durante la gestación, este hallazgo evidencia también la vulneración del derecho fundamental al acceso a servicios sanitarios en mujeres que no tienen capacidad de pago o subsidios del Estado para un seguro de salud (30).

Las desigualdades en el acceso a los servicios odontológicos según el régimen de afiliación, también pueden relacionarse con la forma en que operan y están articulados los diversos sectores en el sistema de salud. En este sentido, en varios estudios se ha evidenciado que la relevancia que dan las mujeres gestantes a su salud bucal y su asistencia efectiva al control odontológico está fuertemente influenciada por la orientación y la remisión que se hace desde los servicios de salud (25,26) y por la importancia que los demás profesionales de la salud le dan a la salud bucal (16,31,32).

En general, la limitada remisión, una actitud pasiva de los diferentes integrantes del sistema de salud para promover y orientar la salud bucal durante el periodo gestacional, así como las percepciones y actitudes propias de las mujeres gestantes, pueden llevar a minimizar su importancia y a aumentar las barreras percibidas por ellas para acceder a la atención odontológica (33).

Las desigualdades según la zona de residencia, el nivel educativo y el estrato socioeconómico pueden ser el reflejo de factores sociales distales, como las condiciones socioeconómicas, culturales, ambientales, vitales y laborales (34), cuya intervención pasa por hacer ajustes estructurales en la sociedad, a lo que el sistema de salud solo ha dado respuesta mediante estrategias como la focalización. La evidencia disponible señala que, principalmente en las zonas rurales, existen barreras geográficas para acceder a los servicios odontológicos (35,36), y que el bajo nivel educativo y socioeconómico de las mujeres gestantes se asocia con condiciones de mayor vulnerabilidad que limitan la asistencia y la oportunidad en el acceso a dichos servicios (24).

Según este análisis, el control odontológico prenatal sigue siendo limitado y se presentan desigualdades en su prestación y su uso. Dado que el sistema de salud es un factor social determinante en sí mismo y debe estar orientado a reducir las inequidades sociales, facilitar el acceso a los servicios sanitarios y generar estrategias en diferentes niveles que respondan a las realidades y necesidades en salud de los diferentes grupos sociales (34), estos resultados pueden aportar elementos de reflexión para incidir sobre dichas desigualdades.

Este estudio tiene algunas limitaciones. Primera, pudo haber sesgos de memoria en los participantes al entregar la información en el momento de la encuesta, sin embargo, el cuestionario fue probado y validado. Segunda, los métodos implementados en la medición de las desigualdades sociales son de tipo bivariado y sencillos, y se aplicaron al análisis a nivel individual, por lo que podrían generar confusión (mezcla de efectos), aunque constituye una ventaja que la información se capturó en el marco de un estudio poblacional, el cual garantiza un diseño estadístico riguroso y una amplia representatividad de la población de interés, por lo que se podrían hacer análisis multivariados adicionales para profundizar en estos resultados.

En conclusión, se evidenció la reproducción de desigualdades en la atención odontológica de las mujeres gestantes, las cuales limitan la garantía del derecho a la salud y la maternidad saludable. Para superarlas, la atención odontológica debe prestarse en el marco de la atención prenatal integral. Los servicios de salud deben promover acciones para lograr una mayor visibilidad de la salud bucal e implementar estrategias diferenciales que mejoren el acceso de las mujeres gestantes a los servicios odontológicos, reduciendo la vulnerabilidad ocasionada por otros factores sociales determinantes.

Es vital reconocer en la praxis que la atención integral e integradora de la maternidad ofrece la oportunidad a los servicios de salud de mejorar el diálogo entre los profesionales, las mujeres y las comunidades y facilita las acciones que inciden en los factores sociales determinantes indispensables para reducir las inequidades en salud y avanzar en la garantía del derecho fundamental a una maternidad saludable.
