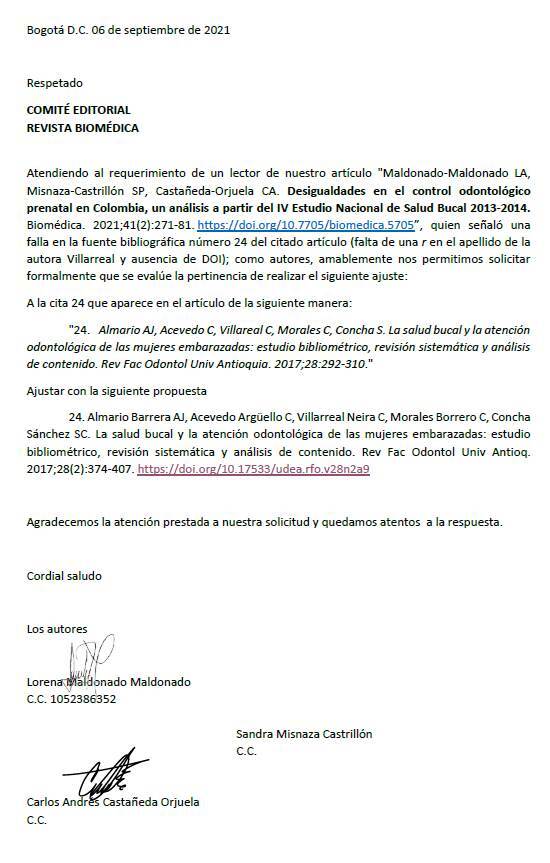# Corrección a los autores

**Published:** 2021-09-22

**Figure:**